# Motivation and learning strategies as predictors of academic maladjustment among first-year engineering students

**DOI:** 10.3389/fpsyg.2026.1946367

**Published:** 2026-09-10

**Authors:** Gabriela Monica Biriș, Ana-Maria Cazan, Simona Nicoleta Neagu

**Affiliations:** 1 Romanian Academy, Institute of Philosophy and Psychology “Constantin Rădulescu-Motru”, Bucharest, Romania; 2 The Department of Teacher Training and Social Sciences, The National University of Science and Technology POLITEHNICA Bucharest, Bucharest, Romania; 3 Faculty of Psychology and Education Science, Transilvania University of Brașov, Brașov, Romania

**Keywords:** academic maladjustment, academic motivation, first-year students, higher technical education, learning strategies

## Abstract

**Introduction:**

This study investigates the relationships between academic motivation, learning strategies, and academic maladjustment among first-year undergraduate students at a science and technology university, with the aim of understanding factors that influence the transition to the university environment.

**Methods:**

A total of 383 first-year students from the National University of Science and Technology POLITEHNICA Bucharest participated in the study. Data were collected online using questionnaires assessing reasons for attending university, learning strategies, and academic maladjustment, including procrastination, test anxiety, lack of honesty, Machiavellian attitudes, neuroticism, and somatization. Correlational analyses and multiple regression analyses were conducted to examine the relationships between these variables.

**Results:**

Expectations and Lack of Alternatives/Inertia emerged as consistent predictors of academic maladjustment, whereas Personal-Intellectual Development, Career/Materialism, and Humanitarian orientations showed limited independent effects. Learning strategies made a relatively small contribution to maladjustment after controlling for motivation. When examined independently, concrete/practical strategies were negatively associated with maladjustment, whereas memorization was positively associated with maladjustment, although these associations were weak.

**Discussion:**

The findings suggest that academic motivation may play a more important role than learning strategies in the academic adjustment of first-year students. The results support the value of integrated interventions combining motivation assessment, counseling, and the development of effective learning strategies to support students’ autonomy, academic adjustment, and social integration.

## 1 Introduction

The transition from secondary education to higher education is an important stage in academic and personal development, characterized by significant changes in cognitive demands, autonomy, and the responsibility for managing one’s own learning. In this context, academic adjustment is considered not only the ability to achieve academic success, but also a multidimensional process involving cognitive, motivational, behavioral, and emotional adjustments to new university demands (Clinciu and Cazan, 2014). Students who successfully adapt develop self-regulated learning strategies, maintain high levels of engagement and academic motivation (Vieriu and Neagu, 2024) and are more likely to persist in their university studies and experience higher levels of academic satisfaction and well-being (Credé and Niehorster, 2012; Richardson et al., 2012).

Academic adjustment is the result of the interaction between a student’s individual characteristics and the educational environment. Recent research highlights the important role of self-efficacy, emotional regulation, self-regulated learning, and academic motivation in facilitating the transition to college (Kritikou and Giovazolias, 2022). At the same time, successful adjustment depends on how higher education institutions respond to the diversity of prior educational experiences and the needs of different student groups. Thus, academic adjustment should be understood as a dynamic process shaped by the interaction between the student’s personal resources and the characteristics of the university context.

These challenges are amplified in engineering fields, where students are exposed to a curriculum characterized by a high cognitive load, complex mathematical and scientific content, experimental activities, and interdisciplinary projects. Engineering education involves not only the acquisition of specialized knowledge but also the development of advanced problem-solving skills, analytical thinking, and independent learning. According to Cognitive Load Theory, the integration of complex and interdependent information can exceed students’ available cognitive capacity, especially when they have not yet developed effective strategies for processing and self-regulating their learning (Sweller et al., 2019). Therefore, first-year engineering students tend to experience adjustment difficulties associated with a high workload, frequent assessments, and the need to quickly adopt effective learning methods.

The diversity of prior academic preparation is an additional factor that influences this transition. Students enter college with varying levels of foundational knowledge, educational experiences, self-regulation skills, and confidence in their own academic abilities. In STEM fields, differences between pre-university preparation and university requirements can lead to increased adjustment difficulties, affecting motivation, engagement, and academic persistence (Sithole et al., 2017). Therefore, identifying the factors that contribute to the successful adjustment of engineering students is *a priori*ty for educational research.

One of the most important psychological determinants of academic adjustment is motivation. According to Self-Determination Theory (Deci and Ryan, 1980; Ryan and Deci, 2017), the quality of motivation influences how individuals engage in activities, persevere in the face of difficulties, and regulate their goal-oriented behaviors. In a university setting, not only is the level of motivation relevant, but so is its nature. Autonomous motivation is based on personal interest, internalized values, and a sense of competence, being associated with deeper engagement and better academic outcomes than predominantly controlled or external forms of motivation. However, the existing literature has focused on motivation as a predictor of academic performance, paying less attention to the difference between the reasons that drive the choice of a field of study and the reasons that sustain subsequent participation and persistence in the university environment. In engineering education, the initial choice of field may be influenced by external factors, such as family expectations, career opportunities, or the social status of the profession, and long-term success depends on the development of internalized and self-regulated motivation.

This perspective is consistent with the model of student development proposed by Côté and Levine (1997), according to which the college experience is a developmental process in which identity, motivation, and socio-contextual influences continuously interact. Although this model provides a relevant theoretical framework for understanding student participation and development, its empirical application in STEM fields and in particular, in engineering education, remains limited.

A second important factor in academic adjustment is the way students learn. Research on self-regulated learning shows that effective students use cognitive and metacognitive strategies to plan, monitor, and evaluate the learning process (Pintrich, 2004; Zimmerman, 2002). The model proposed by Vermunt (1998) expands on this perspective by conceptualizing learning styles as integrated systems that include cognitive processing strategies, regulatory strategies, and motivational orientations. However, few studies have investigated how these complex learning patterns contribute to the academic adjustment of students in demanding engineering programs.

Based on these limitations in the literature, the present study investigates the predictive role of motivation in the choice of and participation in engineering and technology programs, as well as the role of learning styles in the academic adjustment of first-year students. By integrating the motivational perspective of the Self-Determination Theory and the cognitive-regulatory perspective on learning, the study proposes a comprehensive explanatory model of academic adjustment in engineering education. This approach can contribute to a better understanding of the mechanisms through which students respond to high academic demands and can inform the development of student-centered university interventions designed to support retention and academic success.

Building on Self-Determination Theory and the Côté–Levine Input–Process–Output framework, the present study examined whether reasons for attending university and learning strategies predict academic maladjustment among first-year engineering students. We tested three hypotheses:

*H1:* Reasons for attending college predict academic maladjustment, with Expectations and Lack of Alternatives/Inertia expected to show stronger positive predictors of maladjustment than Personal-Intellectual Development, Humanitarian, and Career/Materialism orientations.

*H2:* Learning strategies predict academic maladjustment, with concrete/practical and deep strategies expected to be negatively, and memorization/surface strategies positively, associated with maladjustment.

*H3:* Learning strategies mediate the relationship between reasons for attending college and academic maladjustment.

### 1.1 Academic adjustment

Baker and Siryk’s (1984, 1999) model is one of the most widely cited frameworks for understanding how students adapt to college, distinguishing between academic, social, and emotional adjustment, as well as students’ sense of attachment to their institution. The academic dimension reflects how students respond to educational demands through engagement, time management, the use of learning strategies, sustained effort, and their perception of their own ability to cope with academic tasks, highlighting that success depends not only on prior knowledge, but on the student’s capacity to adapt behaviors and strategies to a new educational context.

The importance of academic adjustment for college success is supported by numerous studies. Through a comprehensive meta-analysis, Credé and Niehorster (2012) demonstrated that adjustment to college is associated with academic performance, satisfaction with the college experience, and persistence in studies. Similarly, Petersen et al. (2009) found that adjustment to university was associated with academic performance among disadvantaged students, highlighting the importance of students’ ability to adapt to the demands of the university environment. Thus, academic adjustment can be considered both the result of university integration, and also a mechanism through which students develop the resources necessary to manage academic demands and reduce the risk of dropping out.

In contemporary models, academic adjustment is considered the result of the interaction between a student’s individual characteristics and the specific features of the educational environment. Research indicates that factors such as academic motivation, self-efficacy, learning strategies, and self-regulation skills significantly contribute to how students adapt to college (van Rooij et al., 2018; Willems et al., 2021). Particularly during the first year of college, these personal resources can facilitate the transition to an environment characterized by higher academic demands and increased responsibility for one’s own learning.

Beyond motivational factors, the ability to self-regulate learning represents a second central mechanism of academic adjustment, examined in detail in the following section. The importance of these mechanisms becomes even more evident in the field of engineering, where students face programs characterized by high conceptual complexity, advanced mathematical and scientific requirements, and tasks that require the integration of multiple types of knowledge. From the perspective of Cognitive Load Theory, adjustment difficulties can arise when the cognitive demands of the educational environment exceed the student’s available resources or when the student lacks effective strategies for managing complex information (Sweller et al., 2019).

First-year engineering students are a relevant group for the study of academic adjustment, as they must simultaneously make the transition to university-level independence, develop problem-solving skills, and adopt new ways of learning. Research in the field of STEM education shows that early academic experiences play an important role in determining students’ persistence and subsequent success (Sithole et al., 2017). Furthermore, studies conducted among engineering students indicate that psychological variables and first-year experiences can significantly contribute to explaining academic performance and retention (García-Ros et al., 2019). Therefore, the literature highlights the fact that academic adjustment is a complex construct resulting from the interaction between motivation, self-regulated learning, and the characteristics of the university environment. However, further research is still needed to simultaneously analyze how motivation specific to choosing and participating in engineering programs, together with students’ learning styles, contributes to academic maladjustment during the first year of college.

### 1.2 Motivation for attending college as a predictor of academic adjustment

Motivation is one of the fundamental psychological mechanisms that explains how students initiate, maintain, and regulate academic behaviors during their transition to higher education. In a university setting, motivation influences not only the level of effort invested but also how students interpret academic challenges, select learning strategies, and develop a sense of belonging within the educational environment.

Contemporary research emphasizes that both the type of motivation and its intensity are determining factors for academic adjustment and long-term success (Ryan and Deci, 2020). According to Self-Determination Theory, motivation can be described on a continuum ranging from autonomous forms to forms controlled by behavioral regulation. Autonomous motivation includes intrinsic motivation and identified regulation and reflects engagement in activities because they are perceived as interesting, valuable, or consistent with personal goals. In contrast, controlled motivation involves participation driven by external pressures, rewards, obligations, or social expectations, while amotivation reflects a lack of intention or a perceived lack of control over one’s behavior (Ryan and Deci, 2020).

In higher education context, autonomous forms of motivation, particularly those centered on personal development, have consistently been associated with deeper academic engagement, greater persistence, the use of adaptive learning strategies, enhanced psychological adjustment, and higher levels of academic satisfaction and achievement (Froiland and Worrell, 2016). Conversely, controlled or poorly internalized forms of motivation, such as external pressures or the perceived lack of alternative educational pathways, are associated with poorer academic adjustment, greater emotional distress, slower academic progress, and an increased likelihood of university dropout (Janke, 2020; Korhonen et al., 2017; Baik et al., 2019; Stanica et al., 2024).

However, in the context of engineering education, academic motivation must be analyzed not only as a general orientation toward learning, but also as a developmental process linked to the reasons why students choose and continue to pursue a particular field of study. Empirical studies on students’ choice of engineering indicate that this decision is shaped by a combination of intrinsic, professional, and social motives. Intrinsic motives include interest in science and technology, identification with engineering, perceived competence in mathematics and science, and a preference for scientific problem-solving and technically oriented activities (Dubreta and Miloš, 2017). Engineering identity and students’ identification with mathematics and physics have also been shown to play an important role in predicting the choice of engineering as a career, with interest and recognition from others contributing to the development of these identities (Godwin et al., 2016). At the same time, extrinsic and career-related motives include employment opportunities, expected income, professional status, and family or social influences (Martin and Sorhaindo, 2019). Students entering engineering programs may therefore have different motivations: interest in science and technology, engineering identity and perceived competence in STEM subjects, a preference for solving scientific and technical problems, personal and intellectual development, professional aspirations, perceived opportunities in the labor market, family influences, or expectations concerning professional status and social recognition. These initial motivations can influence how students relate to academic demands and whether they succeed in developing a sustained commitment to their university studies.

Côté and Levine (1997) argue that university participation is a developmental process in which individual motivations, identity formation, and socio-contextual conditions interact to influence a student’s educational trajectory. Unlike approaches that treat motivation as a stable individual trait, this model considers academic engagement as the result of a progressive process of identity formation and the development of personal goals (Côté and Levine, 2002). The reasons for attending college can be conceptualized as motivational constellations that reflect different student orientations, such as personal and intellectual development, career orientation and material benefits, external influences, or a lack of educational alternatives (Côté and Levine, 1997). These motivational orientations may take particular forms in engineering education, where students’ decisions can also be shaped by engineering identity, perceived competence in mathematics and science, interest in technical problem-solving, and expectations regarding professional opportunities and status.

Empirical research supports this perspective, demonstrating that autonomous motivation is positively associated with academic engagement, self-regulated learning, and educational outcomes (Froiland and Worrell, 2016; Girelli et al., 2018). Students who perceive their college studies as relevant to their personal and professional development tend to use more effective cognitive strategies, manage their time better, and demonstrate greater resilience in the face of academic challenges (Adams and Blair, 2019; Kryshko et al., 2020; Neagu and Vieriu, 2025). In contrast, motivational orientations based predominantly on external pressures or a lack of clear personal goals may be associated with difficulties in adapting and a higher likelihood of academic disengagement.

Although the Côté–Levine motivational orientations can be conceptually interpreted in relation to Self-Determination Theory, they do not constitute direct measures of the autonomous–controlled regulation continuum. Accordingly, SDT is used in the present study as an interpretive framework rather than as a classification system for the five reasons for attending college. Expectations and Lack of Alternatives/Inertia reflect comparatively more externally constrained or poorly self-endorsed reasons, whereas Personal-Intellectual Development is conceptually closer to self-endorsed motivation. Career/Materialism and Humanitarian orientations are retained as distinct Côté–Levine dimensions and are not classified a priori as either autonomous or controlled forms of motivation.

Individual differences in motivation for attending college are particularly important during the transition to college. Studies show that students enter higher education with varying levels of motivational readiness, influenced by prior experiences, social support, career goals, and perceptions of their own future (Dennis et al., 2005; Korhonen et al., 2017). These differences can influence how students respond to academic demands and their ability to adapt to the new educational environment.

In the case of engineering programs, the choice of a specific field and persistence may reflect distinct motivational processes. The initial decision to enroll may be driven by external factors, such as career prospects or social expectations, but long-term academic success requires the development of a professional identity, the internalization of academic goals, and active participation in the learning community. Research in engineering education highlights that engineering identity, a sense of belonging, and professional goals are important predictors of student engagement and persistence (Godwin and Kirn, 2020; Burleson et al., 2021).

Therefore, motivation for attending college can be considered an important predictor of academic adjustment, as it influences how students interpret the college experience, invest effort, and develop strategies for overcoming difficulties. Although a substantial body of research has examined academic motivation in relation to engagement, learning strategies, achievement, and persistence (Froiland and Worrell, 2016; Girelli et al., 2018; Adams and Blair, 2019; Kryshko et al., 2020), relatively less attention has been devoted to the distinction between students’ initial reasons for choosing a particular field of study and the motivational processes underlying their subsequent participation and persistence. In the field of engineering, investigating this distinction can contribute to a deeper understanding of the mechanisms through which students successfully adapt to the demands of their first year of college.

### 1.3 Learning strategies as predictors of academic adjustment

A second major predictor of academic adjustment is represented by the strategy’s students use to manage the demands of learning. Unlike secondary education, university-level learning requires students to organize information independently, regulate their study behavior, monitor their understanding, and adapt their strategies according to the demands of the task. These requirements are more pronounced in engineering education, where students must master complex conceptual systems and apply their knowledge to new situations.

To assess learning strategies, we used Vermunt’s (1994) Inventory of Learning Styles (ILS), a widely adopted instrument in European higher education research. Unlike approaches that treat learning style as a fixed trait, the ILS captures how students characteristically combine cognitive processing strategies, regulatory strategies, learning orientations, and conceptions of learning––defined by Vermunt and Donche (2017) as *learning patterns*. For the purposes of this study, we focused specifically on the cognitive processing and self-regulation dimensions of the ILS, as these are most directly relevant to how students manage academic demands.

Educational psychology literature distinguishes between deep processing, oriented toward understanding and integrating information, and surface processing, based on memorization and reproduction (Vermunt and Donche, 2017; Vermunt and Vermetten, 2004). Deep learning strategies are generally associated with better academic performance and more active engagement in the learning process (Dent and Koenka, 2016; Richardson et al., 2012), although some studies suggest that surface-level strategies may be useful in certain specific contexts (Dinsmore and Alexander, 2012).

From the perspective of self-regulated learning theory, learning strategies are an integral part of the processes through which students plan, monitor, and regulate their cognitive and motivational activity (Panadero, 2017). Self-regulated learning is positively associated with both academic performance and academic adjustment (Cazan, 2012; Hurtado et al., 2007; van Rooij et al., 2018). In contrast, a lack of self-regulation is associated with adjustment difficulties, maladaptive perfectionism, and low performance (Donche et al., 2010; Vieriu and Hainagiu, 2025).

Furthermore, in digitally mediated or hybrid educational contexts, the use of effective learning strategies and self-regulation becomes even more important for academic adjustment, as it is associated with higher levels of cognitive performance and engagement (Broadbent and Poon, 2015; Leonte et al., 2026). Vermunt and Donche (2017) argue that learning patterns offer a more dynamic perspective on student learning by integrating cognitive and regulatory processes. Meaning-oriented learning patterns, characterized by deep processing and self-regulation, are generally associated with adaptive learning behaviors, whereas reproduction-oriented and undirected patterns are more frequently linked to difficulties in adapting to higher education.

Empirical evidence from engineering education supports the relevance of learning styles to student success. Research conducted on first-year engineering students has also shown that study approaches and learning processes are associated with engagement and academic performance, suggesting that adjustment depends not only on students’ knowledge but also on how they regulate their learning activities. Research on self-regulated learning has consistently shown that effective learners actively regulate their cognitive, motivational, and behavioral processes through planning, monitoring, and reflection (Zimmerman, 2002; Panadero, 2017). Students who use deep processing strategies, metacognitive regulation, and organized approaches to learning generally demonstrate higher levels of academic engagement and better adjustment, while surface-level approaches and insufficient regulation are associated with academic difficulties.

In the context of Romanian higher education, research using Vermunt’s Inventory of Learning Styles has shown that learning styles are significant dimensions of university learning, and that active, meaning-oriented learning emerges as an important component of adaptive learning behavior (Cazan and Stan, 2015). However, studies examining the predictive relationship between learning styles and academic adjustment among first-year engineering students remain limited.

### 1.4 Relationship between academic motivation, learning strategies, and adjustment––an integrative perspective

Building on the frameworks reviewed above, an integrative perspective on academic adjustment positions motivation and learning strategies as complementary mechanisms rather than independent predictors. Baker and Siryk (1984) demonstrated that academic adjustment predicts academic performance, persistence, and help-seeking behavior. This body of evidence positions academic adjustment not only as an outcome of university entry, but as an active process through which students develop the motivational and strategic resources needed to persist and succeed. Within this perspective, motivation and learning strategies operate as complementary mechanisms of academic adjustment - motivation providing the direction and persistence for academic engagement, while strategies determine how students translate that motivation into effective learning behaviors (Pintrich, 2004; Zimmerman, 2002). Pintrich and De Groot (1990) showed that motivational beliefs - including self-efficacy, intrinsic value, and test anxiety - significantly predict students’ use of cognitive and metacognitive learning strategies as well as self-regulation. Similarly, the Motivated Strategies for Learning Questionnaire (MSLQ) developed by Pintrich et al. (1991) conceptualizes motivation and learning strategies as complementary dimensions of successful academic functioning, suggesting that motivation influences achievement primarily through the strategic regulation of learning.

Contemporary research has extended these theoretical assumptions by examining how motivational and behavioral variables predict academic adjustment. van Rooij et al. (2018) demonstrated that intrinsic motivation, academic self-efficacy, self-regulated study behavior, and satisfaction with programme choice exert significant positive effects on first-year academic adjustment, which subsequently predicts academic achievement and persistence. Similarly, Willems et al. (2021) reported that motivation, academic self-efficacy, and strategic learning behaviors significantly contribute to students’ successful adjustment across different higher education contexts.

Although prior academic achievement remains an important predictor of university performance, evidence indicates that cognitive ability alone cannot fully explain successful adjustment to higher education. Reviews conducted by Richardson et al. (2012) and Credé and Niehorster (2012) concluded that motivational characteristics, self-regulation, learning strategies, and psychosocial variables explain substantial additional variance in academic performance beyond traditional cognitive indicators. These findings reinforce the importance of considering both cognitive and non-cognitive factors when explaining students’ adjustment to university.

An appropriate theoretical framework integrating these relationships is the Input–Process–Output model proposed by Côté and Levine (2002). Within this perspective, students’ motivations for entering university constitute the input, learning strategies and self-regulatory behaviors represent the learning processes, and academic adjustment emerges as the primary outcome of the interaction between these components. Rather than representing independent predictors, motivation and learning strategies operate sequentially and reciprocally throughout students’ adjustment to higher education.

What remains underexplored, however, is how these motivational and strategic factors operate together among first-year engineering students specifically. Engineering programs are demanding in ways that go beyond typical academic pressure - high dropout rates, heavy workloads, and a steep transition from secondary school mathematics to university-level content make this population relevant for studying adjustment. Understanding which motivational orientations and learning strategies matter most in this context could help institutions identify vulnerable students early and design targeted support.

## 2 Materials and methods

### 2.1 Participants

The study sample consisted of 383 first-year students enrolled at the POLITEHNICA National University of Science and Technology in Bucharest. Data collection took place during the first semester of the academic year, approximately 3 months after the start of classes, a period considered critical for the academic adjustment process. Of the total participants, 209 (54.6%) were male, 167 (43.6%) were female, 3 (0.8%) identified as non-binary, and 4 (1.0%) preferred not to answer. The average age of the participants was 19.06 years (SD = 1.20, range: 18–39 years). Participants were recruited on a voluntary basis. The study was approved by the Scientific Research Ethics Committee of the POLITEHNICA National University of Science and Technology in Bucharest (Approval No. 221/10/15/2025).

### 2.2 Instruments

Students’ motivations for attending college were measured using the Student Motivations for College Attendance Questionnaire (Côté and Levine, 1997). The 23 items are measured on a six-point Likert scale, ranging from “strongly disagree” to “strongly agree,” and are grouped into five scales: the Careerism-Materialism (CAR) motivation scale (5 items, α = 0.87) measures motives related to viewing college as a means of earning money and status; examples of items include: “College is the way I will end up having an interesting and satisfying career, and college is a concrete way for me to achieve personal success.”

Personal-Intellectual Development (PER) (5 items, α = 0.84) highlights the role of the university in personal development; example item: “It is satisfying to attend college because here I have the opportunity to study and learn; “College provides me with an environment where I can grow personally.” The Humanitarian Motivation Scale (HUM) (4 items, α = 0.86) emphasizes the role of attending college in helping those less fortunate and changing the world; example item: “I intend to ensure that what I study contributes to the well-being of others.” The Expectation-Driven Motivation Scale (EXP) (5 items, α = 0.78) involves expectations and pressures from family and friends to attend college: “I am a student primarily because I expect to earn a degree”. The Lack of Alternatives/Inertia dimension (DEF) (4 items, α = 0.76) reflects unclear reasons for attending university, including a perceived lack of viable alternatives or inertia (e.g., “I am a university student because I did not really have other alternatives.”).

Learning strategies were assessed using the Inventory of Learning Styles (Vermunt, 1994), a widely used tool in European educational research. The ILS conceptualizes learning as a multidimensional process that includes cognitive and self-regulatory strategies.

In this study, three types of strategies were analyzed: Memorization and repetition—a surface-level strategy focused on reproducing information; Concrete/applied learning—an applied strategy focused on the practical use of knowledge; and Analogy/associations—a deep learning strategy. An example of an item: “I try to identify the connection between the topics discussed in different chapters of the course.”

Responses are rated on a 5-point Likert scale (ranging from “rarely or never” to “almost always”). Internal consistency coefficients (Cronbach’s alpha) were also calculated, indicating adequate to good reliability for all scales and subscales:

The Deep Information Processing Scale, which includes the analogies and associations subscale (7 items, α = 0.86); example item: “I try to connect new information to what I already know about the topic”; The Sequential Processing Scale, with the memorization and Repetition subscale (5 items, α = 0.83); example item: “I repeat the main points until I learn them by heart” and the Concrete/practical Learning Scale (5 items, α = 0.79), example item: “When I’m learning a topic in class, I think of examples from my own experience that are related to that topic.”

Academic maladjustment was assessed using the Academic Maladjustment Questionnaire (CIA; Clinciu and Cazan, 2014; Cazan et al., 2023), which was developed and validated among the student population in Romania. The instrument conceptualizes academic maladjustment as a multidimensional construct, including affective dimensions: Academic neuroticism (α = 0.85); Test anxiety (α = 0.92); behavioral dimensions: Procrastination (α = 0.89); Academic dishonesty (α = 0.90); Machiavellian attitude (α = 0.81); and Somatization (α = 0.79). Items are rated on a Likert scale, with higher scores indicating a higher level of academic maladjustment. Example items: “I am unable to organize either my time or my schoolwork” or “When I am called on to answer a question in class, I turn pale, stutter, or have trouble formulating my thoughts.” The instrument demonstrates predictive validity, particularly in relation to the risk of dropping out of college.

### 2.3 Procedure

Data collection was conducted in collaboration with faculty members using an online questionnaire administered via Google Forms as part of students’ educational activities. Participants were informed about the purpose of the study, the voluntary nature of participation, and the confidentiality and anonymity of their responses. Before accessing the questionnaire, participants provided electronic informed consent through Google Forms. The average completion time was approximately 20–25 min. The study was conducted in accordance with the ethical principles set forth in the Declaration of Helsinki (World Medical Association, 2013) and was approved by the Scientific Research Ethics Committee of the POLITEHNICA National University of Science and Technology in Bucharest (Approval No. 221/10/15/2025).

### 2.4 Data analysis

The data were analyzed using quantitative statistical methods: correlation analyses (Pearson) to examine the relationships between variables; multiple linear regressions to test predictive hypotheses; and, to examine whether learning strategies mediate the relationship between motivation and academic maladjustment, a parallel mediation analysis with bootstrapping was conducted. In the regression and mediation analyses, the dependent variable was a composite academic-maladjustment score, computed as the mean of the six CIA subscale scores (procrastination, academic dishonesty, test anxiety, machiavellian attitude, neuroticism, and somatization). This composite showed high internal consistency (Cronbach’s α = 0.92 across the 30 items).

The assumptions of regression (normality, linearity, multicollinearity) were verified, and the coefficients were interpreted using 95% confidence intervals.

All analyses were conducted in IBM SPSS Statistics (v.29). Descriptive statistics, reliability analyses (Cronbach’s α), Pearson correlations, and the multiple linear regressions were computed in SPSS. The parallel multiple-mediation analysis was estimated with the PROCESS macro (Hayes, Model 4) using 5,000 bootstrap samples and bias-corrected 95% confidence intervals.

## 3 Results

### 3.1 Relationships between motivation to attend college, learning strategies, and academic maladjustment

To examine the relationships between motivation to attend college, learning strategies, and indicators of academic maladjustment, descriptive statistics and Pearson correlation coefficients were calculated (Table 1).

**Table 1:** Descriptive statistics and Pearson correlation matrix for the study variables (*N* = 383).

Variable	M	SD	1	2	3	4	5	6	7	8	9	10	11	12	13	14
1. Career/Materialism	4.56	1.12	—													
2. Personal-Intellectual Dev.	4.54	1.08	0.54***	—												
3. Humanitarian	4.22	1.35	0.42***	0.55***	—											
4. Expectations	2.71	1.44	0.21***	0.14**	0.17***	—										
5. Lack of Alternatives/ Inertia	1.48	1.30	−0.17***	−0.24***	−0.12*	0.37***	—									
6. Memorization/ repetition	2.90	0.88	0.14**	0.16**	0.13**	0.04	−0.08	—								
7. Concrete/ practical	3.13	0.79	0.03	0.19***	0.14**	−0.05	−0.18***	0.06	—							
8. Analogy/ associations	3.63	0.71	0.08	0.19***	0.10	−0.02	−0.13*	0.09	0.57***	—						
9. Procrastination	2.33	0.69	0.03	0.02	0.04	0.33***	0.38***	−0.08	−0.18***	−0.17**	—					
10. Academic dishonesty	1.78	0.63	−0.04	−0.17***	−0.07	0.12*	0.40***	−0.04	−0.18***	−0.19***	0.37***	—				
11. Test anxiety	2.11	0.95	0.05	0.03	0.11*	0.31***	0.24***	0.09	−0.07	−0.08	0.42***	0.26***	—			
12. Machiavellian attitude	2.06	0.73	0.08	−0.02	−0.03	0.15**	0.30***	0.01	−0.13*	−0.09	0.29***	0.67***	0.18***	—		
13. Neuroticism	1.95	0.92	0.11*	0.13*	0.15**	0.23***	0.20***	0.21***	−0.04	−0.01	0.32***	0.16**	0.53***	0.20***	—	
14. Somatization	2.36	0.86	0.10	0.09	0.12*	0.28***	0.27***	0.10*	−0.09	−0.02	0.58***	0.23***	0.56***	0.26***	0.57***	—

CAR, Career/Materialism; PER, Personal-Intellectual Development; HUM, Humanitarian; EXP, Expectations; DEF, Lack of Alternatives/Inertia; MEM, Memorization/repetition; CONC, Concrete/practical; ANA, Analogy/associations; PROC, Procrastination; NEO, Academic dishonesty; ANX, Test anxiety; MACH, Machiavellian attitude; NEUR, Neuroticism; SOM, Somatization. *p < 0.05; **p < 0.01; ***p < 0.001.

The correlation analysis showed that the Expectations (EXP) and Lack of Alternatives/Inertia (DEF) dimensions were positively associated with the indicators of academic maladjustment, whereas Career/Materialism (CAR), Personal-Intellectual Development (PER), and Humanitarian (HUM) showed weaker or non-significant associations.

Regarding learning strategies, they are predominantly associated with the behavioral dimensions of academic maladjustment (procrastination and academic dishonesty), while the relationships with affective indicators (neuroticism and somatization) are generally weak or insignificant.

Furthermore, Personal-Intellectual Development (PER) was positively associated with concrete/practical learning and analogy/association strategies, whereas Lack of Alternatives/Inertia (DEF) was negatively associated with these strategies.

To test the predictive hypotheses, a hierarchical multiple regression was conducted with the composite academic-maladjustment score as the dependent variable: the five motivation scales were entered in Step 1 and the three learning strategies in Step 2 (Table 2). Step 1 was significant, *R*^2^ = 0.23, *F*(5, 377) = 22.59, *p* < 0.001; Expectations (β = 0.17, *p* < 0.001) and Lack of Alternatives/Inertia (β = 0.37, *p* < 0.001) were the only significant predictors, whereas Career/Materialism, Personal-Intellectual Development and Humanitarian were non-significant––supporting H1. Adding the learning strategies in Step 2 produced a small but significant increment, Δ*R*^2^ = 0.02, *F*(3, 374) = 3.50, *p* = 0.016 (total *R*^2^ = 0.25, adjusted *R*^2^ = 0.24). In the full model, memorization was weakly and positively associated with maladjustment (β = 0.10, *p* = 0.027), whereas concrete/practical (β = −0.07, *p* = 0.19) and analogy/associations (β = −0.06, *p* = 0.30) were not significant. All variance inflation factors were below 1.9, indicating no multicollinearity.

**Table 2:** Hierarchical multiple regression predicting composite academic maladjustment (*N* = 383).

Predictor	*B*	*SE*	*β*	*t*	*p*	95% CI	VIF
CAR	0.030	0.027	0.060	1.08	0.281	[−0.024, 0.084]	1.53
PER	0.019	0.031	0.037	0.61	0.545	[−0.042, 0.080]	1.83
HUM	0.027	0.023	0.065	1.18	0.240	[−0.018, 0.071]	1.51
EXP	0.067	0.020	0.174	3.41	<0.001***	[0.028, 0.106]	1.31
DEF	0.155	0.022	0.364	7.03	<0.001***	[0.112, 0.199]	1.34
MEM	0.064	0.029	0.101	2.22	0.027*	[0.007, 0.121]	1.04
CONC	−0.051	0.039	−0.073	−1.31	0.190	[−0.127, 0.025]	1.53
ANA	−0.045	0.043	−0.057	−1.04	0.298	[−0.129, 0.039]	1.49

Full model: *R*^2^ = 0.252, adjusted *R*^2^ = 0.236, *F*(8, 374) = 15.71, *p* < 0.001. Step 1 (motivation) *R*^2^ = 0.23; Δ*R*^2^ (adding strategies) = 0.02, *F*(3, 374) = 3.50, *p* = 0.016. *p < 0.05. **p < 0.01. ***p < 0.001.

These results support Hypothesis H1, highlighting the predominant role of Expectations and Lack of Alternatives/Inertia in predicting academic maladjustment. Regarding learning strategies as predictors of academic maladjustment (H2) and when they were entered on their own, without motivation, a multiple linear regression analysis was conducted. The model was statistically significant, but its explanatory power was modest *F* (3, 379) = 5.14, *p* = 0.002, *R*^2^ = 0.039. Only the concrete/practical learning strategy was a significant predictor of academic maladjustment (*B* = −0.092, *p* = 0.024), whereas memorization and repetition and analogy and associations were not significant predictors. However, this effect did not remain significant once motivation was controlled in the full model (Table 2); accordingly, H2 received only weak and partial support.

To examine the mediating role of learning strategies in the relationship between motivation and academic maladjustment (H3), a parallel mediation analysis was conducted using the bootstrapping procedure. Three mediating variables were simultaneously included in the model: memorization and repetition strategies (MEM), concrete/practical strategies (CONC), and strategies based on analogies and associations (ANA). The analysis was conducted using the bootstrapping procedure with 5,000 bootstrap subsamples. The statistical significance of the indirect effects was assessed using 95% bootstrap confidence intervals (Bias-Corrected 95% Confidence Intervals). The analyses were performed in IBM SPSS Statistics, using the PROCESS macro developed by Andrew F. Hayes (Model 4––parallel mediation).

**Figure 1:**
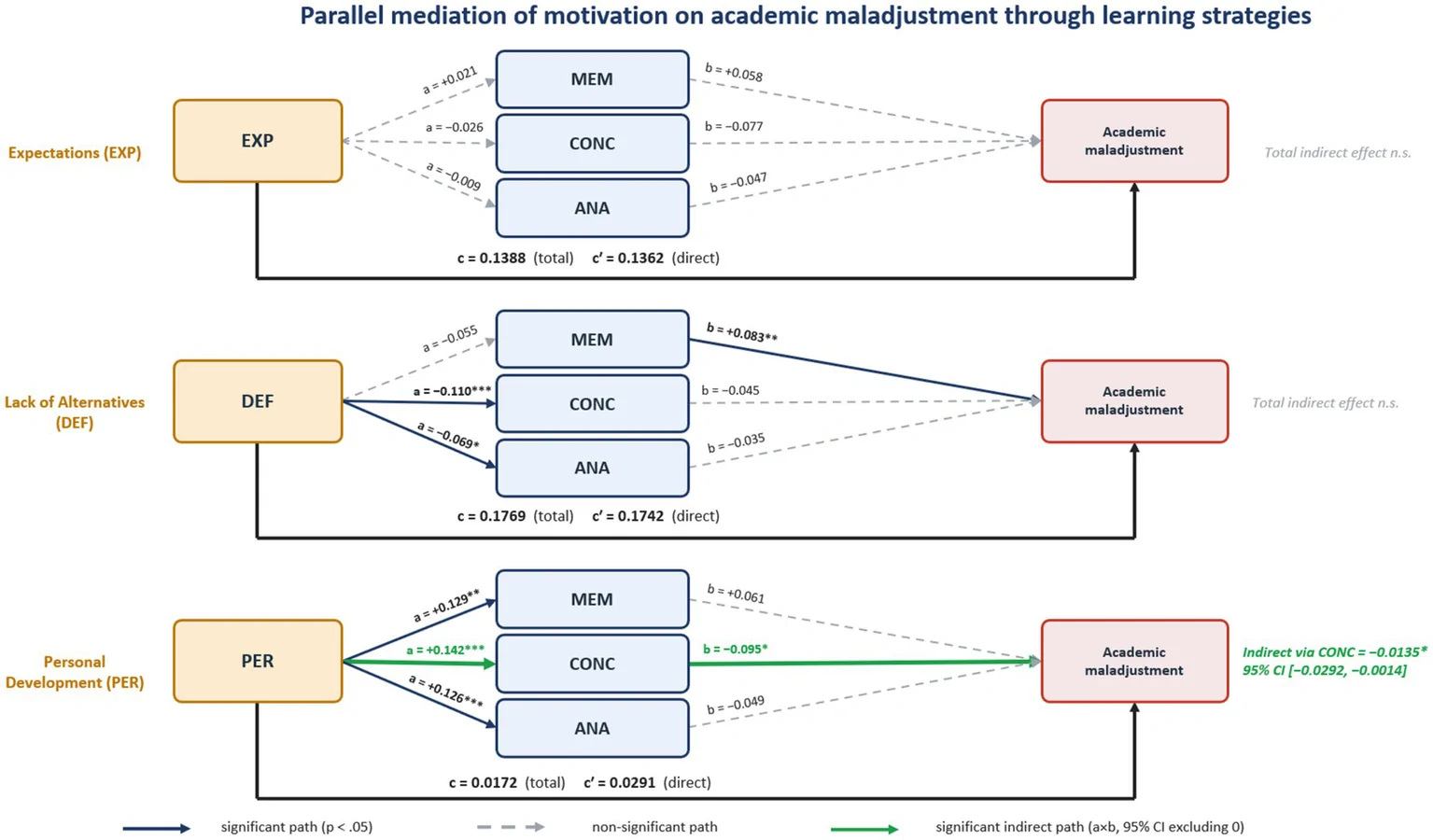
Parallel mediation model of the relationship between motivation for attending university and academic maladjustment through learning strategies (Separate parallel-mediation models per predictor; in each, the three mediators (MEM = memorization, CONC = concrete/practical, ANA = analogy/associations) were entered simultaneously (not sequentially). *a* = predictor → mediator; b = media tor → outcome; indirect = a × *b*; *c* = total effect, *c′* = direct effect. Unstandardized coefficients; bootstrap 5,000 samples, bias-corrected 95% CIs. *N* = 383).

The parallel mediation of motivation on academic maladjustment through learning strategies is presented in Figure 1. Three separate parallel-mediation models were estimated, one for each motivational predictor (Expectations, EXP; Lack of Alternatives/Inertia, DEF; Personal Development, PER); in every model the three learning strategies (memorization, MEM; concrete/practical, CONC; analogy/associations, ANA) were entered simultaneously as parallel mediators, not sequentially. Each panel displays the *a* paths (predictor → mediator), the *b* paths (mediator → outcome), the total effect *c* and the direct effect *c′*. Solid arrows denote statistically significant paths (*p* < 0.05) and dashed arrows non-significant paths; the green pathway marks the only complete indirect route that reached significance. Because an indirect effect is the product of its two components (indirect = *a* × *b*), a mediating pathway is meaningful only when both *a* and *b* are significant. This condition was met only for Personal Development through concrete/practical strategies (PER → CONC → maladjustment; *a* × *B* = −0.0135, 95% bias-corrected bootstrap CI [−0.0292, −0.0014], 5,000 resamples). For EXP and DEF, at least one link in every chain was non-significant, so no indirect effect emerged, and all total indirect effects were non-significant. Consistent with this, the total effects of EXP and DEF on maladjustment were substantial and largely direct (*c* = 0.1388 and 0.1769; *c′* = 0.1362 and 0.1742, respectively), whereas the effect of PER was small and non-significant overall (*c* = 0.0172) and operated almost entirely through the indirect route rather than directly (*c′* = 0.0291, *n.s.*). Coefficients are unstandardized (N = 383).

The results of the parallel mediation analysis are presented in Table 3. For the dimensions Expectations (EXP) and Lack of Alternatives (DEF), neither the specific indirect effects nor the total indirect effects were statistically significant, as the 95% bootstrap confidence intervals included zero. Furthermore, the small differences between the total and direct effects suggest that including learning strategies in the model does not alter the relationship between these motivational dimensions and academic maladjustment. These results indicate that the influence of expectations and the perception of a lack of alternatives on academic maladjustment is predominantly direct and is not explained by the learning strategies investigated.

**Table 3:** Indirect effects (parallel mediation) of motivation on academic maladjustment through learning strategies.

Predictor	Mediator (M)	B indirect	95% CI	Total effect (c)	Direct effect (c′)
Expectations (EXP)	Memorization (MEM)	0.0004	[−0.0042, 0.0052]	0.1388	0.1362
	Concrete/practical (CONC)	0.0021	[−0.0016, 0.0080]		
	Analogy/associations (ANA)	0.0000	[−0.0037, 0.0039]		
	Total indirect	0.0026	[−0.0049, 0.0104]		
Lack of alternatives (DEF)	Memorization (MEM)	−0.0046	[−0.0125, 0.0013]	0.1769	0.1742
	Concrete/practical (CONC)	0.0049	[−0.0038, 0.0155]		
	Analogy/associations (ANA)	0.0024	[−0.0035, 0.0107]		
	Total indirect	0.0027	[−0.0088, 0.0143]		
Personal development (PER)	Memorization (MEM)	0.0078	[−0.0006, 0.0197]	0.0172	0.0291
	Concrete/practical (CONC)	−0.0135	[−0.0292, −0.0014]		
	Analogy/associations (ANA)	−0.0062	[−0.0218, 0.0057]		
	Total indirect	−0.0119	[−0.0340, 0.0066]		

Bias-corrected 95% confidence intervals, 5,000 bootstrap samples. No total indirect effect was significant. Only the Personal Development → Concrete/practical specific path was significant (CI excludes zero). Decimal points standardized to periods.

A different pattern was observed for the Personal and Intellectual Development (PER) dimension.

Of the three learning strategies included in the model, only the concrete/practical strategy (CONC) showed a significant indirect effect (*B* = −0.0135, 95% CI [−0.0292, −0.0014]). The negative sign of the indirect effect indicates that a personal development orientation is associated with more frequent use of concrete and applied strategies, which, in turn, are associated with lower levels of academic maladjustment.

In contrast, the indirect effects estimated using the memorization and repetition (MEM) and analogy and associations (ANA) strategies were not statistically significant, as their confidence intervals included zero. Furthermore, the total indirect effect of personal development did not reach the threshold for statistical significance (*B* = −0.0119, 95% CI [−0.0340, 0.0066]), suggesting that the mediating mechanism does not operate at the level of learning strategies as a whole, but rather through a specific pathway involving concrete/practical learning.

Overall, none of the three motivational dimensions showed a significant total indirect effect on academic maladjustment. Only one specific indirect effect, from Personal-Intellectual Development through concrete/practical learning strategies, reached significance. Because this specific effect occurred within a non-significant total indirect effect, it should be interpreted cautiously and does not provide robust evidence of mediation. Taken together, H3 was largely not supported: the associations of motivation with academic maladjustment were predominantly direct rather than mediated by learning strategies.

## 4 Discussions

### 4.1 Academic motivation and university adjustment

This study examined the relationship between motivation to participate in college, learning strategies, and academic maladjustment among first-year college students. Overall, the results indicate that academic adjustment is primarily influenced by the quality of the motivation underlying engagement in academic activities, while learning strategies play a secondary explanatory role, contingent on the motivational context.

This pattern is consistent with recent literature, which highlights that the success of the transition to higher education depends largely on the internalization of the reasons for attending college and the quality of self-regulation that these reasons support (Kritikou and Giovazolias, 2022; Howard et al., 2021).

The main finding of the study is the association between Expectations and Lack of Alternatives/Inertia and higher levels of academic maladjustment. These orientations reflect comparatively more externally constrained or poorly self-endorsed reasons for attending university and were associated with procrastination, academic dishonesty, test anxiety, neuroticism, and somatization, suggesting a more vulnerable adaptive profile. This pattern can be interpreted in light of Self-Determination Theory, according to which controlled or poorly internalized reasons for action are associated with difficulties in self-regulation and psychological functioning (Deci and Ryan, 2000; Ryan and Deci, 2020). At the same time, they are consistent with the review conducted by Kritikou and Giovazolias (2022) and the meta-analysis by Howard et al. (2021), which highlight the relationship between controlled motivation, academic stress, integration difficulties, and less favorable academic and psychological outcomes. The results of the present study extend these conclusions, suggesting that the effects of poorly defined and undirected motivation are reflected in a broader spectrum of academic maladjustment.

The interpretation of these results is also supported by the theoretical model of learning-related demands and resources, according to which adjustment depends on the balance between the demands of the academic environment and the student’s personal resources (Bakker and Mostert, 2024). From this perspective, externally constrained or poorly self-endorsed reasons for attending university may reduce the availability of resources necessary for adjustment and promote stress and maladaptive coping strategies.

Previous research suggests that the effects of more self-determined forms of motivation may operate indirectly through self-regulation and engagement in learning (Ryan and Deci, 2020; Howard et al., 2021). Research on student engagement shows that the relationship between autonomous motivation and academic success is mediated by cognitive and behavioral engagement in learning activities (Fredricks et al., 2019; Rothes et al., 2022). In the present study, Personal-Intellectual Development was positively associated with more adaptive learning strategies; however, as discussed below, the mediation analyses provided only limited evidence that these strategies transmitted its association with academic maladjustment.

The results support and expand upon the model of academic integration proposed by Tinto (1975), highlighting that college adjustment depends not only on commitment to one’s studies, but especially on the quality of the motivation that underpins it. In line with both Tinto’s (1975) integration model and Bean’s (1980) attrition model, the finding that Expectations and Lack of Alternatives/Inertia predict higher maladjustment suggests that poorly internalized reasons for attending university may represent an early risk factor for withdrawal. Overall, the findings suggest that Expectations and Lack of Alternatives/Inertia represent particularly relevant motivational risk factors for academic maladjustment, whereas the other motivational orientations examined in this study did not show significant direct predictive effects. This pattern can be interpreted within SDT while recognizing that the Côté–Levine dimensions are not direct measures of autonomous and controlled regulation.

### 4.2 The role of learning strategies and mediation analysis

A second focus of this study was the role of learning strategies in the relationship between academic motivation and academic maladjustment. Although self-regulated learning theory regarding motivation posits that cognitive and metacognitive strategies are the mechanisms through which motivation influences academic outcomes (Zimmerman, 2002; Panadero, 2017), our results indicate that this role is selective, depending on both the type of strategy and the student’s motivational profile.

The mediation analysis provided little support for the hypothesized mediating role of learning strategies. None of the three motivational dimensions showed a significant total indirect effect on academic maladjustment. For Personal-Intellectual Development, one specific indirect path through concrete/practical learning strategies reached significance; however, given the non-significant total indirect effect, this isolated finding should be interpreted cautiously. Overall, H3 was largely not supported, indicating that the associations between reasons for attending college and academic maladjustment were predominantly direct rather than mediated by the learning strategies examined in this study.

These results are consistent with Vermunt’s (1998) model, according to which motivational orientations influence the choice of cognitive strategies and styles of information processing. Along the same lines, Howard et al. (2021) and Rothes et al. (2022) show that autonomous motivation promotes the use of elaboration strategies and cognitive engagement, while controlled motivation is more frequently associated with superficial approaches to learning.

Furthermore, the absence of a mediating role for memorization and repetition strategies suggests that they primarily facilitate the reproduction of information without contributing significantly to academic adjustment finding consistent with the literature on self-regulated learning (Panadero, 2017; Howard et al., 2021). Although Personal-Intellectual Development was associated with more adaptive learning strategies, the mediation analyses did not provide robust evidence that these strategies mediated its association with academic maladjustment (Ryan and Deci, 2020).

At the same time, the absence of indirect effects for Expectations and Lack of Alternatives/Inertia suggests that their associations with academic maladjustment may involve other mechanisms, such as academic anxiety, burnout, frustration of fundamental psychological needs, or reduced engagement-mechanisms also highlighted in recent literature on adjustment to higher education (Hyytinen et al., 2022; Kritikou and Giovazolias, 2022; Howard et al., 2021).

The findings complement the basic principles of self-determination theory and self-regulated learning theory, suggesting that the effectiveness of learning strategies depends on the motivational context and that their development is unlikely to produce lasting effects in the absence of strengthened autonomous motivation.

### 4.3 Study’s contribution and theoretical and practical implications

This study contributes to the literature on academic adjustment supporting a model in which motivation is the primary determinant of adjustment, learning strategies play a secondary role, with only limited and non-significant mediating effects, thereby contributing to the literature on self-regulated learning (Vermunt, 1998; Panadero, 2017; Howard et al., 2021).

The study also makes a methodological contribution through its multidimensional conceptualization of academic maladjustment. Unlike studies that use a single indicator of adjustment, such as academic performance or intention to drop out, the present study simultaneously integrates behavioral indicators (procrastination and academic dishonesty) and psychological indicators (test anxiety, neuroticism, somatization, and Machiavellian attitude), offering a more comprehensive perspective on the difficulty’s students face during the transition to higher education (Cazan et al., 2023).

From a practical perspective, the results suggest that interventions targeting first-year students should combine support for autonomous motivation with the promotion of deep processing strategies, the clarification of academic goals, and the development of a sense of competence. In line with the literature on student retention and the academic demands and resources model, such an approach can facilitate adjustment to the university environment and reduce the risk of adjustment difficulties and dropout (Bakker and Mostert, 2024; Hyytinen et al., 2022; Ahmad and Akram Rana, 2024).

Overall, the study proposes an integrative model of academic adjustment that highlights the interdependence between motivation and self-regulation and provides a useful conceptual framework for both future research and the development of interventions aimed at improving student adjustment and retention.

### 4.4 Limitations, future research directions, and conclusions

Several limitations should be discussed. The cross-sectional design does not allow for causal inferences regarding the relationships between academic motivation, learning strategies, and academic maladjustment. Although the proposed model is supported by the literature on self-determination theory and self-regulated learning (Ryan and Deci, 2020; Panadero, 2017), longitudinal studies are needed to track the evolution of these relationships during the transition to higher education.

In addition, the exclusive use of self-report instruments may introduce common method errors and social desirability effects. Future research could incorporate objective indicators of academic adjustment, such as academic performance, college retention, or faculty evaluations, to gain a more comprehensive perspective on the adjustment process.

Another limitation stems from the characteristics of the sample, which consists exclusively of first-year students. Although this stage is critical for university integration (Tinto, 1975; Kritikou and Giovazolias, 2022), generalizing the results to other categories of students or institutional contexts should be done with caution. Furthermore, the moderate explanatory power of the models suggests that academic adjustment is a multifactorial process. Future research should include variables such as academic self-efficacy, engagement, the fulfillment of basic psychological needs, social support, and the educational climate - factors identified in recent literature as important predictors of student adjustment and retention (Howard et al., 2021; Bakker and Mostert, 2024).

Despite these limitations, the study provides additional evidence regarding the role of academic motivation in explaining the adjustment of students at the beginning of their college journey. The results indicate that Expectations and Lack of Alternatives/Inertia are directly associated with higher levels of academic maladjustment, while motivation oriented toward personal development was associated with maladjustment mainly at the direct level, with only a small, non-significant indirect contribution via learning strategies.

Overall, the results outline an integrative model of academic adjustment in which the quality of motivation directly influences adjustment and learning strategies play only a limited role, since their mediating (indirect) effects were small and non-significant. These findings expand the literature on studies addressing self-determination theory and self-regulated learning and provide arguments for the development of university interventions aimed simultaneously at supporting autonomous motivation and fostering self-regulation.

## 5 Conclusion

The present study highlights the central role of reasons for attending university in explaining first-year students’ academic maladjustment. Expectations and Lack of Alternatives/Inertia were consistently associated with higher levels of academic maladjustment, whereas Personal-Intellectual Development, Career/Materialism, and Humanitarian orientations did not show significant direct predictive effects. The mediation analyses provided little support for an indirect role of learning strategies, as all total indirect effects were non-significant.

These findings suggest that learning strategies are not universal mechanisms of academic adjustment but operate selectively depending on students’ motivational profiles. Consequently, interventions designed to facilitate the transition to higher education should combine the promotion of autonomous motivation with the development of deep learning and self-regulated learning strategies. By integrating the perspectives of Self-Determination Theory and Self-Regulated Learning, this study provides evidence that fostering students’ internal motivational resources may represent a key pathway for improving academic adjustment and supporting long-term student retention in higher education.

## Data Availability

The original contributions presented in the study are included in the article/supplementary material, further inquiries can be directed to the corresponding author.
